# Pyroptosis in pterygium pathogenesis

**DOI:** 10.1042/BSR20180282

**Published:** 2018-05-22

**Authors:** Naiyu Sun, Hong Zhang

**Affiliations:** The First Affiliated Hospital of Harbin Medical University, Harbin, Heilongjiang Province, People’s Republic of China

**Keywords:** caspase-1, IL-1β, oxidative stress, NLRP3, pterygium, pyroptosis

## Abstract

Pterygium is a common ocular disease characterized by proliferating fibrovascular tissue. Pyroptosis, a recently discovered programed cell death, is known to be associated with oxidative stress, one of the main causes of pterygia. Here, we aimed to study the role of pyroptosis in pterygium pathogenesis. The expression of nod-like receptor pyrins-3 (NLRP3), caspase-1, IL-18, and IL-1β was analyzed in 60 human pterygium tissues and 60 human conjunctival epithelium tissues using real-time quantitative polymerase chain reaction (qRT-PCR) and Western blot analysis. Human conjunctival epithelial cells (HConECs) and human pterygium fibroblasts (HPFs) were primary cultured and the level of pyroptosis-associated factors was detected. Both cells were treated with H_2_O_2_, and cell lysis was detected by lactate dehydrogenase (LDH) release assay, the expression of the factors by qRT-PCR, Western blot analysis, and immunostaining. The downstream factors IL-18 and IL-1β were measured after inhibition of caspase-1 to confirm the caspase-1-dependent pyroptosis. α-SMA and E-cadherin were detected as indicators of pyroptosis-induced myofibroblast activation in HPFs. We discovered that the expression of the factors was significantly increased in pterygium and that caspase-1-dependent pyroptosis presents in both H_2_O_2_-treated HPFs and HConECs during which the expression of these factors was significantly elevated and the elevation of downstream factors IL-18 and IL-1β was restrained after caspase-1 inhibition. α-SMA increase and E-cadherin down-regulation were detected in H_2_O_2_-treated HPFs and the changes were reversed by caspase-1 inhibition. Pyroptosis displays a role in the pathological process of pterygium formation and progression. Pyroptosis appears to be an intriguing target to prevent pterygium pathogenesis.

## Introduction

Pterygium is an ocular surface disorder characterized as a wing-like shape on the nasal limbus, which hinders vision, causes inflammation, and affects cosmetic appearance.

Limbal stem cell degeneration and dissolution of Bowman’s membrane occur in the histopathology of pterygium formation, accompanied by activated fibroblast growth, neovascularization, excessive proliferation of extracellular matrix, and inflammation [1].

Surgery is still the main treatment for pterygia, although it has a high risk of surgical complications such as recurrence, graft necrosis, and granuloma formation [2]. The recurrence after surgery is still common, although diverse efforts have been made to avoid it [3]. Therefore, understanding the mechanism of pterygium formation is of vital importance in improving treatment and decreasing occurrence rate.

Multiple pathogenic factors, including UV light exposure [4], virus infection [5], oxidative stress [6], fibrosis, and cell epithelial–mesenchymal transition [5], inflammation cascade [7,8], apoptosis [9], extracellular matrix modulators [10], DNA methylation [11], angiogenic, and lymphangiogenic stimulation [12,13], have been proposed, yet the exact mechanisms of pterygium formation have not been elucidated.

Pyroptosis, a proinflammatory programed cell death [14] with the feature of rapid plasma-membrane rupture and release of proinflammatory intracellular contents [15], can be triggered by various pathological stimuli such as microbial infections [16], cardiovascular diseases [17], central nervous system diseases [18], and malignant tumors [19]. Nod-like receptor pyrins-3 (NLRP3), caspase-1, IL-18, and IL-1β are commonly accepted markers of pyroptosis. Several pathogen-associated molecular patterns (PAMPs), accompanied by damage-associated molecular patterns (DAMPs) initiate the process by activating NLRP3 [20,21]. NLRP3 is the initiating pyroptosis factor responding to certain cellular stress signals triggered by PAMPs and DAMPs. The activated NLRP3, under the oligomerization with apoptosis-associated speck-like protein (ASC), compromises as NLRP3 inflammasome which facilitates the proteolytic cleavage of pro-caspase-1 to activated caspase-1. Only after the activation of the caspase-1-dependent secretion mechanism can the procytokines be cleaved into mature IL-1β and IL-18 and secreted from cytosol and successively provoke a series of reactions [22,23].

The accumulation of reactive oxygen species (ROS) is an extensively studied model that can cause NLRP3 activation [24]. Oxidative stress is also one of the pathogenic factors in the formation of pterygium [25]. However, whether pyroptosis participates in the initiation and progression for pterygia remains unknown. In the present study, we characterized the expression of NLRP3, caspase-1, IL-18, and IL-1β in pterygia compared with normal human conjunctival epithelium. We also explored pyroptosis involvement in pterygium formation and verified its effects on human conjunctival epithelial cells (HConECs) as well as human pterygium fibroblasts (HPFs). Our data established for the first time that pyroptosis plays a role in the genesis and development of pterygia.

## Materials and methods

### Materials

Dulbecco’s modified Eagle’s medium/F12 (DMEM/F12), Dulbecco’s modified Eagle’s medium/low glucose (DMEM/low glucose), and fetal bovine serum (FBS) were obtained from BI (Israel). H_2_O_2_ was purchased from Shanghai Zhongshi Chemistry Industry Co (Shanghai, China); Cell Counting Kit-8 was purchased from Dojindo Laboratories (Shanghai, China), and CytoTox 96 Cytotoxicity Assay from Promega Corporation (U.S.A.); caspase-1 inhibitor Ac-YVAD-cmk was purchased from Cayman Chemical (U.S.A.); TRIzol kit and PCR primers were purchased from Invitrogen (Carlsbad, CA); the concentration of RNA was detected by a NanoDrop Spectrophotometer (NanoDrop Technologies, Wilmington, DE). Primary antibodies (anti-caspase-1, anti-NLRP3, anti-IL-1β, anti-β-actin, anti-α-SMA, and anti-E-cadherin) were purchased from Abcam (Cambridge, MA). Fluorochrome labeled secondary antibody (Alexa Fluor 488, molecular probes, U.S.A.) was used to identify the target primary antibody.

### Human tissue samples

Sixty pterygium samples were collected from pterygium patients free from other ocular diseases in the operating room of the Eye Hospital of Harbin Medical University, China. Sixty control conjunctival epithelium samples (free of ocular diseases) were obtained from healthy donor eyes donated to the eye bank of Heilongjiang Province. Samples were immediately snap-frozen and stored at −80°C until RNA or protein extraction. All samples were collected with informed consent and the study was approved by the Research Ethics Committee of Harbin Medical University.

### HConEC and HPF primary culture and cell treatment

Another five pairs of donated normal human conjunctiva samples and surgically dissected human pterygium tissue samples were preserved in DMEM before transferring to the laboratory. The tissues were washed three times in PBS (containing 500 U/ml penicillin and 500 µg/ml streptomycin) and the conjunctiva epithelial layer/pterygium was carefully separated and disintegrated into 1–2 mm^3^ pieces and seeded in Petri dishes precoated with FBS afterward. Complete culture media was added 8 h later to achieve full attachment of the tissues. The primary cell cultures were incubated at 37°C with 5% CO_2_. The cell cultures were observed daily, and the medium was changed every 3 or 4 days. Subculturing was done when the cells reached 70–80% confluence and were spared into culture flasks containing complete culture media. Five primary cultured cell lines were established each from different patients/donors. Cells in the logarithmic growth phase were collected and co-cultured with various concentrations of H_2_O_2_ for the required time periods. The experiment was performed between passages 3 and 10.

### Cell proliferation assay

Cell proliferation assay kits (CCK-8) were used in accordance with the manufacturer’s instructions. HConECs and HPFs were seeded in 96-well plates, and CCK-8 solution was added after proper treatment; the plate was maintained at 37°C for 4 h before detection. The absorbance at 450 nm was used in the evaluation with a microplate reader.

### Lactate dehydrogenase assay

The cells were plated in 96-well culture dishes and lactate dehydrogenase (LDH) release was detected after stimulation of H_2_O_2_ for 6 h. LDH level in the supernatant was quantitively measured under the instruction of CytoTox 96 Cytotoxicity Assay and asorbance at 490nm was evaluated with a microplate reader. LDH release percentage, indicating the pyroptotic rate, was calculated as the equation: [(experimental release−spontaneous release)/(maximum release−spontaneous release)] ×100, in which spontaneous release represents LDH release from unstimulated cells while maximum release represents unstimulated cells treated with lysis solution in the kit.

### Real-time quantitative polymerase chain reaction

Total RNA was extracted from tissues or cultured cells with TRIzol reagents, and the isolated RNA was reverse-transcribed into synthesized cDNA in a 20μl reaction mixture. Real-time quantitative polymerase chain reaction (qRT-PCR) was performed according to SYBR Green qPCR Master Mix instructions. The primers were synthesized by Co Invitrogen Ltd, Beijing, China and the sequences are listed in Table 1. The housekeeping gene glyceraldehyde-3-phosphate dehydrogenase (GAPDH) was taken as an internal positive control standard for quantitative analysis. Data were analyzed using the 2^−ΔΔC_T_^ method.

**Table 1:** The primers used for qRT-PCR

Primer		Sequence
GAPDH	Forward	5′-AAGAAGGTGGTGAAGCAGGC-3′
	Reverse	5′-TCCACCACCCTGTTGCTGTA-3′
NLRP3	Forward	5′-TTCAATGGCGAGGAGAAGGC-3′
	Reverse	5′-ACGTGTCATTCCACTCTGGC-3′
Caspase-1	Forward	5′-ACACGTCTTGCCCTCATTATCT-3′
	Reverse	5′-ATAACCTTGGGCTTGTCTTTCA-3′
IL-18	Forward	5′-TCTTCATTGACCAAGGAAATCGG-3′
	Reverse	5′-TCCGGGGTGCATTATCTCTAC-3′
IL-1β	Forward	5′-CCTTGTCGAGAATGGGCAGT-3′
	Reverse	5′-TTCTGTCGACAATGCTGCCT-3′
α-SMA	Forward	5′-CCGTGATCTCCTTCTGCATT-3′
	Reverse	5′-CTGTTCCAGCCATCCTTCAT-3′
E-cadherin	Forward	5′-CAAGCTATCCTTGCACCTCAG-3′
	Reverse	5′-GCATCAGAGAACTCCTATCTTG-3′

### Western blot analysis

Western blot analysis was performed to detect the expression of target proteins. The total protein content was measured following the manufacturer’s instructions of BCA Protein Assay Kits. Equal amounts of protein were separated with 10% SDS/PAGE; after which the protein bands were transferred onto the membrane. The membrane was incubated with target primary antibodies overnight at 4°C with gentle shaking. Goat-anti-rabbit secondary antibody was added to identify the appropriate primary antibody. Odyssey fluorescent scanning system (LI-COR) and Quantity One software were used for the detection and analysis of the immunoreactivity. β-actin was introduced as a loading control.

### Immunofluorescence staining

HConECs and HPFs were rinsed with PBS after being fixed with 4% paraformaldehyde at 37°C for 1 h. Cells were blocked with 10% BSA and then incubated with rabbit caspase-1 (1:200; Abcam), NLRP3 (1:300; Abcam), or IL-1β (1:200; Abcam) overnight at 37°C. Corresponding secondary antibody was incubated after rinsing three times with PBS. The nuclei were stained with DAPI.

### Statistical analysis

All data were expressed as the mean ± standard deviation (SD). SPSS 18.0 statistical software was used to perform statistical analysis. Statistical significance was analyzed by Student’s t-test after testing data for normality (according to the K-S test). *P*-value less than 0.05 was considered statistically significant.

## Results

### Exploration of pyroptosis in the pterygium tissues

**Figure 1:**
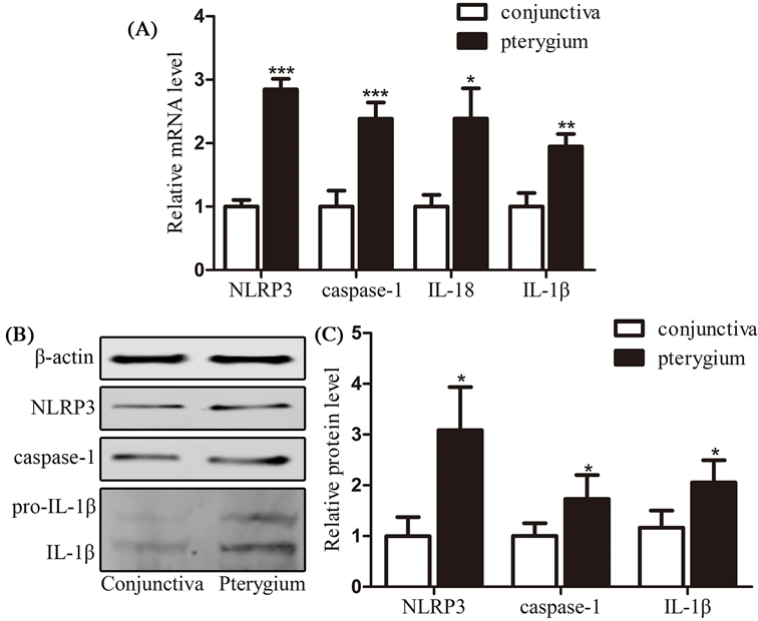
Validation of pyroptosis in the pterygium tissues (**A**) qRT-PCR results of the relative mRNA levels of NLRP3, caspase-1, IL-18, and IL-1β in 30 pairs of normal conjunctiva epithelium and pterygium samples; (**B** and **C**) Western blot analysis of NLRP3, caspase-1, and IL-1β protein levels in another 30 pairs of normal conjunctiva epithelium and pterygium samples. Data are expressed as mean ± SEM; ^*^P<0.05, ^**^P<0.01, and ^***^P<0.001 compared with control.

Thirty pterygium samples and thirty normal conjunctiva samples were collected and analyzed for NLRP3, caspase-1, IL-18, and IL-1β mRNA expression using qRT-PCR (Figure 1A). The mRNA of these factors was highly expressed in pterygium samples with statistical difference (NLRP3: *P*<0.001; caspase-1: *P*<0.001; IL-1β: *P*<0.05; IL-18: *P*<0.01). Another 30 pterygium samples and 30 normal conjunctiva samples were detected for NLRP3, caspase-1, pro-IL-1β, and IL-1β protein level with Western blot analysis (Figure 1B,C). The protein levels of these factors were highly expressed in pterygium samples compared with normal conjunctiva samples with statistical difference (NLRP3, caspase-1, and IL-1β: *P*<0.05). The expression level of pyroptosis-related factors was significantly up-regulated in pterygium samples compared with normal conjunctival epithelial samples, suggesting the existence of pyroptosis in the pterygium tissues.

### Validation of pyroptosis in HPFs

**Figure 2:**
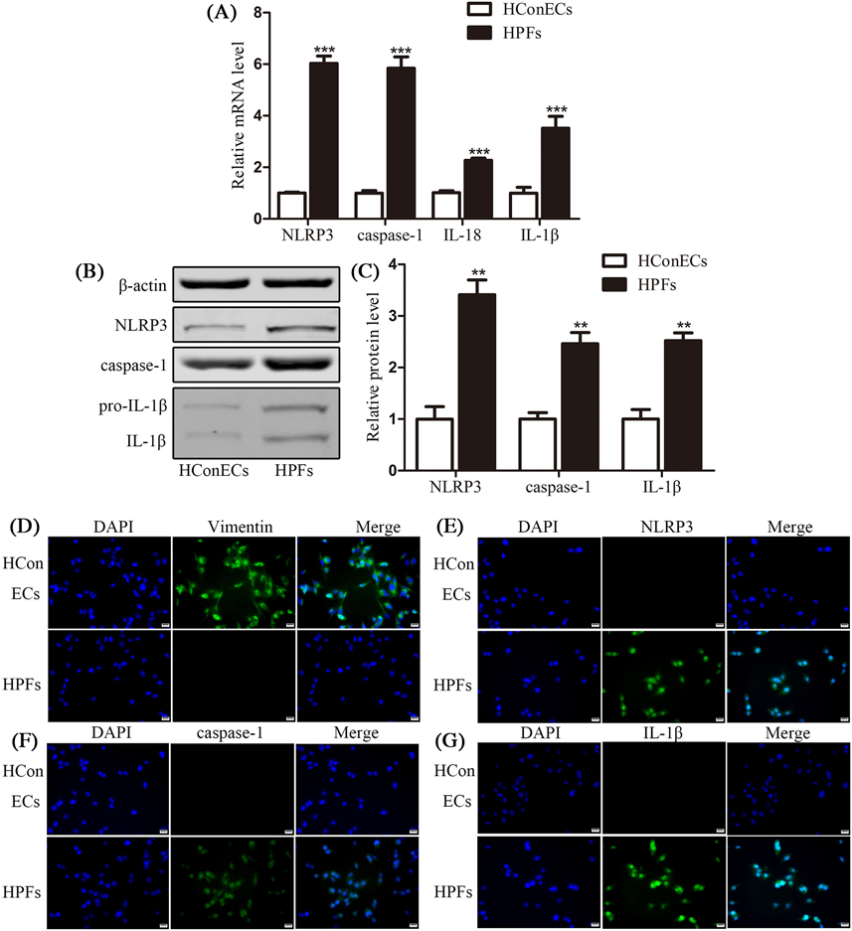
Validation of pyroptosis in HPFs (**A**) qRT-PCR results of the relative mRNA levels of NLRP3, caspase-1, IL-18, and IL-1β in HConECs and HPFs (five primary cultured cell lines each); (**B** and **C**) Western blot analysis of NLRP3, caspase-1, and IL-1β protein levels in HConECs and HPFs (five primary cultured cell lines each); (**D**) immunofluorescence images of vimentin are presented demonstrating the high expression of vimentin in elongated fibroblasts cytosol compared with its minimum expression in epithelial cells; (**E–G**) immunofluorescence images showing the expression of NLRP3, caspase-1, and IL-1β in HConECs and HPFs, respectively. Blue: nuclear staining (DAPI); green: NLRP3, caspase-1, or IL-1β staining. Scale bar: 20 µm; ^*^P<0.05, ^**^P<0.01, and ^***^P<0.001 compared with control.

HConECs and HPFs were primary cultured using the tissue adherent method and identified with immunofluorescence staining of vimentin (Figure 2D). qRT-PCR was performed to analyze the mRNA expression of NLRP3, caspase-1, IL-18, and IL-1β while Western blot analysis and immunofluorescence staining were used to detect protein level of the related factors (Figure 2A–C and E–G). According to the results, HPFs held high levels of NLRP3, caspase-1, IL-18, and IL-1β mRNA expression with *P*<0.001, compared with HConECs. The protein expressions of NLRP3, caspase-1, IL-18, and IL-1β also presented high level in comparison with HConECs with *P*<0.01. Immunofluorescence images showed similar result with Western blot analysis presenting that fluorescent intensity of NLRP3, caspase-1, and IL-1β was higher in HPFs than HConECs. The elevation of pyroptosis-related factors in HPFs provided more proof in validation of pyroptosis in pterygium genesis.

### Pyroptosis participated in H_2_O_2_-treated HConECs

**Figure 3:**
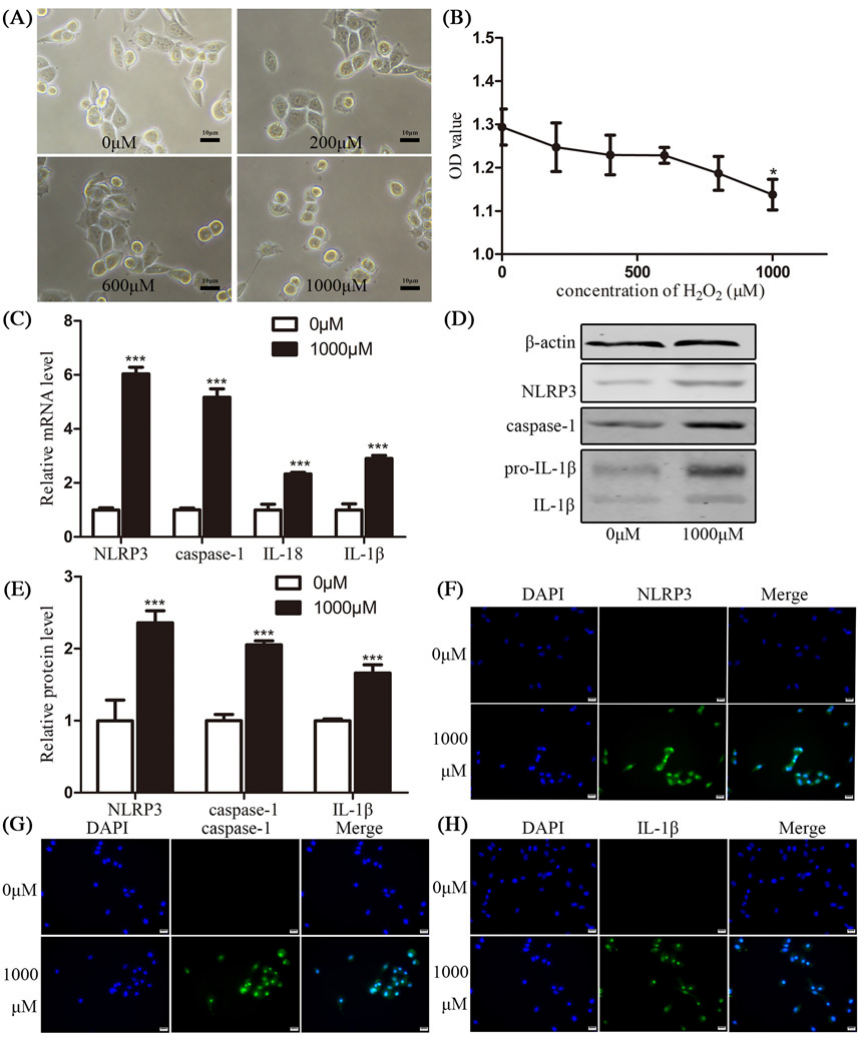
Pyroptosis participated in H_2_O_2_-treated HConECs (**A**) Morphology images presented HConECs after co-culture with H_2_O_2_ (0, 200, 600, and 1000 µM) for 24 h, HConECs became swollen and cell number decreased with the increase of the H_2_O_2_ concentration. Scalebar: 10µm; (**B**) CCK-8 assay results revealed that HConECs’ growth was inhibited when co-cultured with H_2_O_2_ for 24 h; (**C**) qRT-PCR results showed that the relative NLRP3, caspase-1, IL-18, and IL-1β mRNA levels were increased at 1000 µM H_2_O_2_ for 24 h; (**D** and **E**) Western blot analysis revealed that NLRP3, caspase-1, and IL-1β protein levels were up-regulated after stimulation of 1000 µM H_2_O_2_ for 24 h, results are representative of five independent experiments; mRNA and protein expression of these factors under other H_2_O_2_ concentrations are presented in Figures 8 and 9; (**F–H**) immunofluorescence images showing the expression of NLRP3, caspase-1, and IL-1β in HConECs treated with H_2_O_2_ under the concentration of 1000 µM for 24 h (1000 µM) compared with control group (0 µM). Blue: nuclear staining (DAPI); green: NLRP3, caspase-1, and IL-1β staining, respectively. Scale bar: 20 µm; ^*^P<0.05, ^**^P<0.01, and ^***^P<0.001 compared with control.

In order to explore whether pyroptosis is involved in pterygium formation, various concentrations of H_2_O_2_ (0, 200, 400, 600, 800, and 1000 µM) were adopted to stimulate HConECs for 24 h. Pyroptosis was detected by morphology observation, CCK-8, qRT-PCR, Western blotting assays, and immunostaining (Figure 3A–H). After exposure to H_2_O_2_, the viability of HConECs was suppressed according to CCK-8 result, the proliferation of HConECs presented a dose-dependent fashion of inhibition after H_2_O_2_ treatment (Figure 3A-B) and at the concentration of 1000 µM, the inhibition showed statistical difference (*P*<0.05). qRT-PCR results in Figure 3C demonstrated that the relative NLRP3, caspase-1, IL-1β, and IL-18 mRNA levels were significantly increased at 1000 µM H_2_O_2_ for 24 h (*P*<0.001), the mRNA levels were elevated for over 100% compared with control group (HConECs treated with 0 µM H_2_O_2_). Western blot analysis in Figure 3D,E presented that the relative NLRP3, caspase-1, and IL-1β protein levels were increased at 1000 µM H_2_O_2_ for 24 h, with statistical difference (*P*<0.001). The protein levels were elevated for over 50% compared with control group (HConECs treated with 0 µM H_2_O_2_). mRNA and protein level of these factors after being treated with different concentrations of H_2_O_2_ are presented in Figures 8 and 9 and Supplementary Figure S1. Immunofluorescence images (Figure 3F–H) presented that fluorescent intensity of NLRP3, caspase-1, and IL-1β were significantly increased in HConECs after being treated with 1000 µM H_2_O_2_. The elevation of pyroptosis-related factors in H_2_O_2_-treated HConECs indicated that pyroptosis can be induced by H_2_O_2_ and thus participates in pterygium formation.

**Figure 4:**
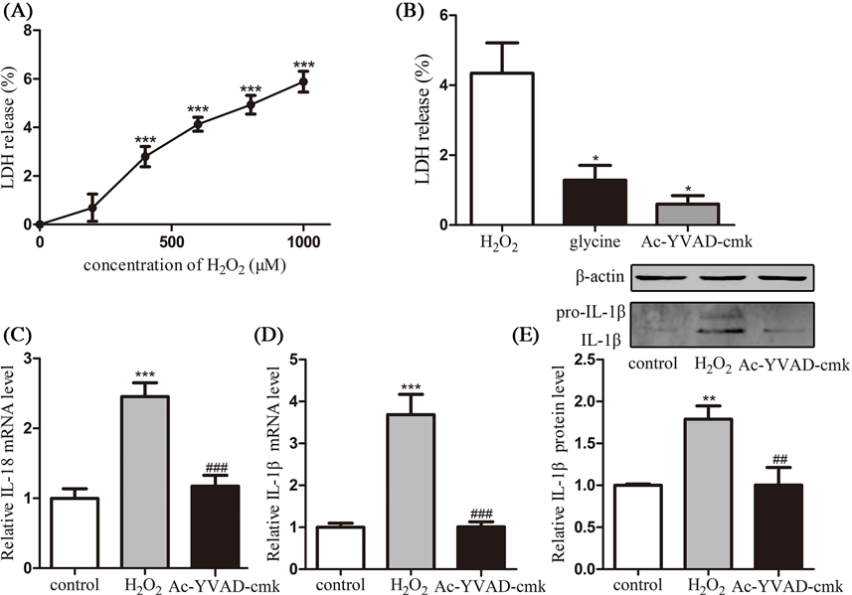
Caspase-1 inhibitor restrained pyroptosis in HConECs caused by H_2_O_2_ (**A** and **B**) LDH release assay presented an H_2_O_2_ dose-dependent LDH release into the supernatant by HConECs after being stimulated for 6 h and the release of LDH at the concentration of 1000 µM was inhibited by glycine as well as Ac-YVAD-cmk (n = 5); (**C** and **D**) relative mRNA levels of IL-18 and IL-1β in untreated HConECs (control group), HConECs co-cultured with 1000 µM H_2_O_2_ for 24 h (H_2_O_2_ group) and 1000 µM H_2_O_2_ + Ac-YVAD-cmk (Ac-YVAD-cmk group); (**E**) relative protein level of IL-1β in control group, H_2_O_2_ group, and Ac-YVAD-cmk group. All results are representative of over three independent experiments; ^*^P<0.05, ^**^P<0.01, and ^***^P<0.001 compared with control group; ^##^*P*<0.01 and ^###^*P*<0.001 compared with H_2_O_2_ group.

In order to confirm the pyroptosis cause by H_2_O_2_, the release of cytosolic LDH in the supernatant after 6 h H_2_O_2_ stimulation was taken as an indicator of cell lysis during pyroptosis. As shown in Figure 4A, LDH release percentage of HConECs also presented a dose-related manner in accordance with H_2_O_2_ concentration. Moreover, as shown in Figure 4B, the cell lysis caused by H_2_O_2_ was significantly protected by glycine (*P*<0.05) and obviously suppressed by caspase-1 inhibitor Ac-YVAD-cmk (*P*<0.05), confirming the caspase-1-dependent cell lysis in H_2_O_2_-treated HConECs. To further confirm the caspase-1-dependent pyroptosis cause by H_2_O_2_, caspase-1 inhibitor Ac-YVAD-cmk was introduced to suppress caspase-1 activation, and downstream targets IL-18 and IL-1β were investigated with qRT-PCR and Western blot. According to Figure 4C–E, the expression of IL-18 and IL-1β was significantly decreased when selective caspase-1 inhibitor Ac-YVAD-cmk was added to 1000 µM H_2_O_2_-treated HConECs.

### Pyroptosis participated in H_2_O_2_-treated HPFs

**Figure 5:**
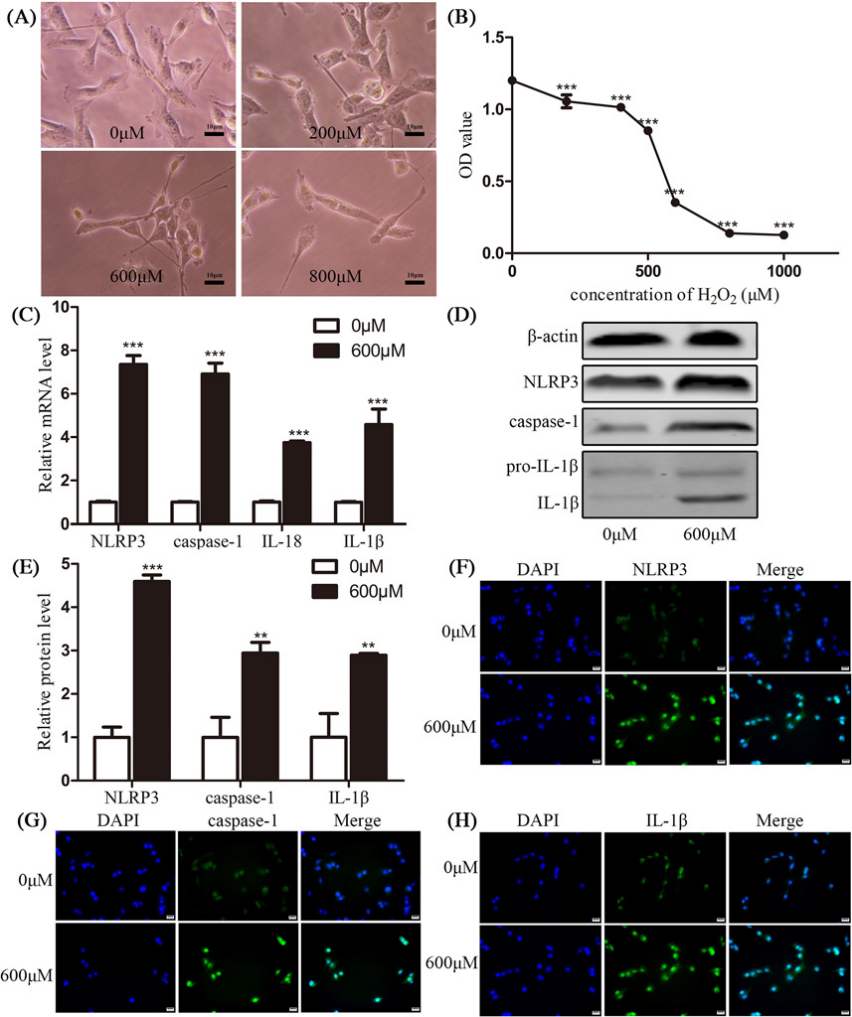
Pyroptosis participated in H_2_O_2_-treated HPFs (**A**) When exposed to various concentrations of H_2_O_2_ (0, 200, 600, and 800 µM) for 24 h, HPFs’ cell number was severely decreased with the increase of the H_2_O_2_ concentration. Scalebar: 10µm; (**B**) CCK-8 assay results revealed that HPFs’ growth was inhibited in a dose-dependent fashion when co-cultured with H_2_O_2_ for 24 h; (**C**) qRT-PCR results showed that the relative NLRP3, caspase-1, IL-18, and IL-1β mRNA levels were increased at 600 µM H_2_O_2_ for 24 h; (**D** and **E**) Western blot analysis revealed that NLRP3, caspase-1, and IL-1β protein levels were up-regulated after stimulation of 600 µM H_2_O_2_ for 24 h, results are representative of five independent experiments; mRNA and protein expression of these factors under other H_2_O_2_ concentrations are extended in Figures 8 and 9; (**F–H**): immunofluorescence images showing the expression of NLRP3, caspase-1, and IL-1β in control group as well as HPFs stimulated with H_2_O_2_ under same condition. Blue: nuclear staining (DAPI); green: NLRP3, caspase-1, and IL-1β staining, respectively. Scale bar: 20 µm; ^*^P<0.05, ^**^P<0.01, and ^***^P<0.001 compared with control.

To detect the function pyroptosis performed in pterygium progression, HPFs were exposed to various concentrations of H_2_O_2_ (0, 200, 400, 600, and 800 µM) for 24 h. H_2_O_2_-induced pyroptosis was detected accordingly (Figure 5A–H). The proliferation of HPFs was inhibited in a dose-dependent fashion with H_2_O_2_ concentration at 24 h and the inhibition started to appear statistically different at 200 µM (*P*<0.001) and the inhibition showed the most marked impact after the concentration of H_2_O_2_ reached 600 µM (Figure 5A,B). When exposed to different concentrations of H_2_O_2_, the mRNA and protein levels of NLRP3, caspase-1, IL-18, and IL-1β were also increased in a dose-dependent manner. Compared with the control group, the relative mRNA and protein level of pyroptosis associated factors were increased over 200% when the H_2_O_2_ concentration exceeded 600 µM (Figure 5 C–H). mRNA and protein level of these factors after being treated with different concentrations of H_2_O_2_ are presented in Figures 8 and 9 and Supplementary Figure S1. Immunofluorescence images presented that fluorescent intensity of NLRP3, caspase-1, and IL-1β were significantly increased in HPFs after being co-cultured with 600 µM H_2_O_2_. The elevation of pyroptosis-related factors in HPFs treated with H_2_O_2_ indicated that pyroptosis can also be induced by H_2_O_2_ in existing pterygium tissues and thus participates in pterygium progression.

**Figure 6:**
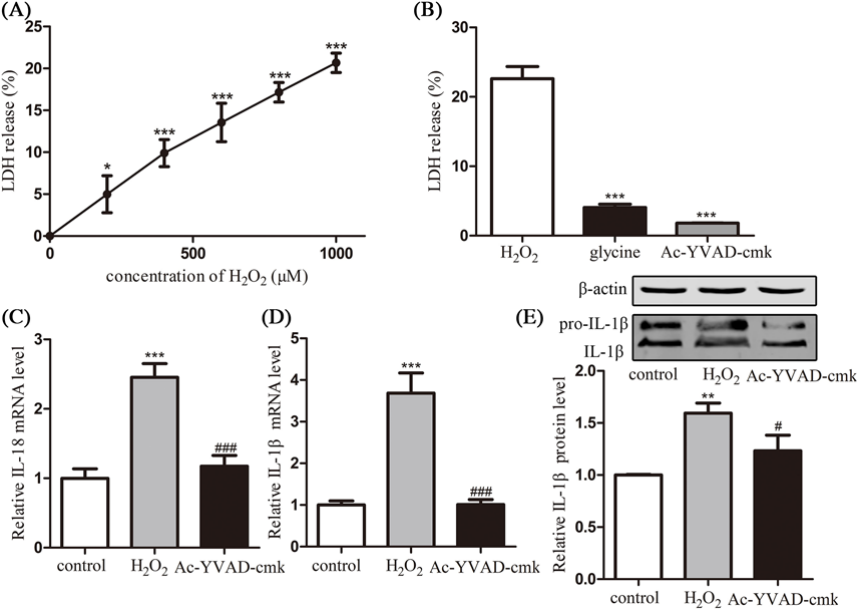
Caspase-1 inhibitor restrained pyroptosis in HPFs caused by H_2_O_2_ (**A** and **B**) LDH release assay presented an H_2_O_2_ dose-dependent LDH release into the supernatant by HPFs after being stimulated for 6 h and the release of LDH at the concentration of 1000 µM was inhibited by glycine as well as Ac-YVAD-cmk (n = 5); (**C** and **D**) relative mRNA levels of IL-18 and IL-1β in untreated HPFs (control group), HPFs co-cultured with 600 µM H_2_O_2_ for 24 h (H_2_O_2_ group) and 600 µM H_2_O_2_ + Ac-YVAD-cmk (Ac-YVAD-cmk group); (**E**) relative protein level of IL-1β in control group, H_2_O_2_ group, and Ac-YVAD-cmk group. All results are representative of over three independent experiments; ^**^P<0.01 and ^***^P<0.001 compared with control group; ^#^*P*<0.05 and ^###^*P*<0.001 compared with H_2_O_2_ group.

For confirmation of the pyroptosis caused by H_2_O_2_ in HPFs, LDH release percentage of HPFs was measured to indicate the relationship between H_2_O_2_ concentration cell lysis level (Figure 6A). As a result, H_2_O_2_-treated HPFs demonstrated a dose-dependent fashion of LDH release. Moreover, the cell lysis caused by H_2_O_2_ was largely restrained by glycine (*P*<0.001) and almost prevented by caspase-1 inhibitor Ac-YVAD-cmk (*P*<0.001), as shown in Figure 6B. Caspase-1 inhibition of 600 µM H_2_O_2_-treated HPFs performed by Ac-YVAD-cmk led to a sharp decrease of downstream IL-18 and IL-1β expression, indicating that the caspase-1-dependent pyroptosis occurs in H_2_O_2_-treated HPFs (Figure 6C–E).

**Figure 7:**
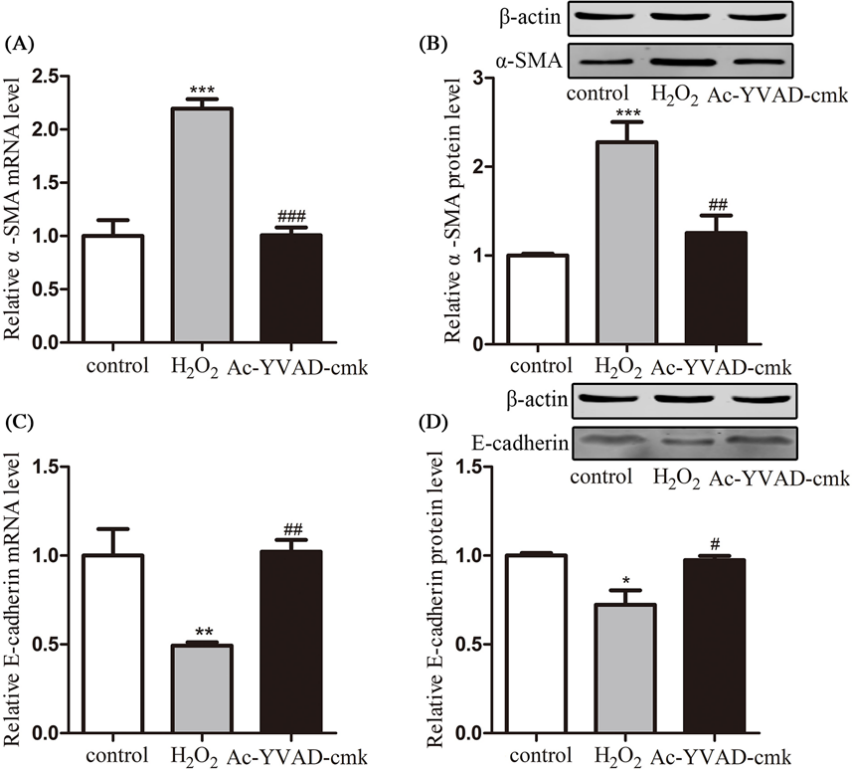
HPF activation and pyroptosis (**A** and **B**) Relative mRNA and protein levels of α-SMA in untreated HPFs (control group), HPFs co-cultured with 600 µM H_2_O_2_ for 24 h (H_2_O_2_ group), and 600 µM H_2_O_2_ + Ac-YVAD-cmk (Ac-YVAD-cmk group); (**C** and **D**) relative mRNA and protein levels of E-cadherin in control group, H_2_O_2_ group, and Ac-YVAD-cmk group. All results are representative of over three independent experiments; ^*^P<0.05, ^**^P<0.01, and ^***^P<0.001 compared with control; ^#^*P*<0.05, ^##^*P*<0.01, and ^###^*P*<0.001 compared with H_2_O_2_ group.

To further investigate the role of pyroptosis of HPFs in pterygium progression, mRNA and protein levels of α-SMA and E-cadherin were detected after H_2_O_2_-related pyroptosis was induced. According to Figure 7A,B, mRNA and protein level of α-SMA was largely elevated after 600 µM H_2_O_2_ stimulation for 24 h and the elevation could be reversed by caspase-1 inhibitor. Similarly, mRNA and protein level of E-cadherin was down-regulated after the same H_2_O_2_ stimulation and the decrease was significantly prevented by caspase-1 inhibitor (Figure 7C,D).

### Comparison of pyroptosis factors in HConECs and HPFs after stimulation

**Figure 8:**
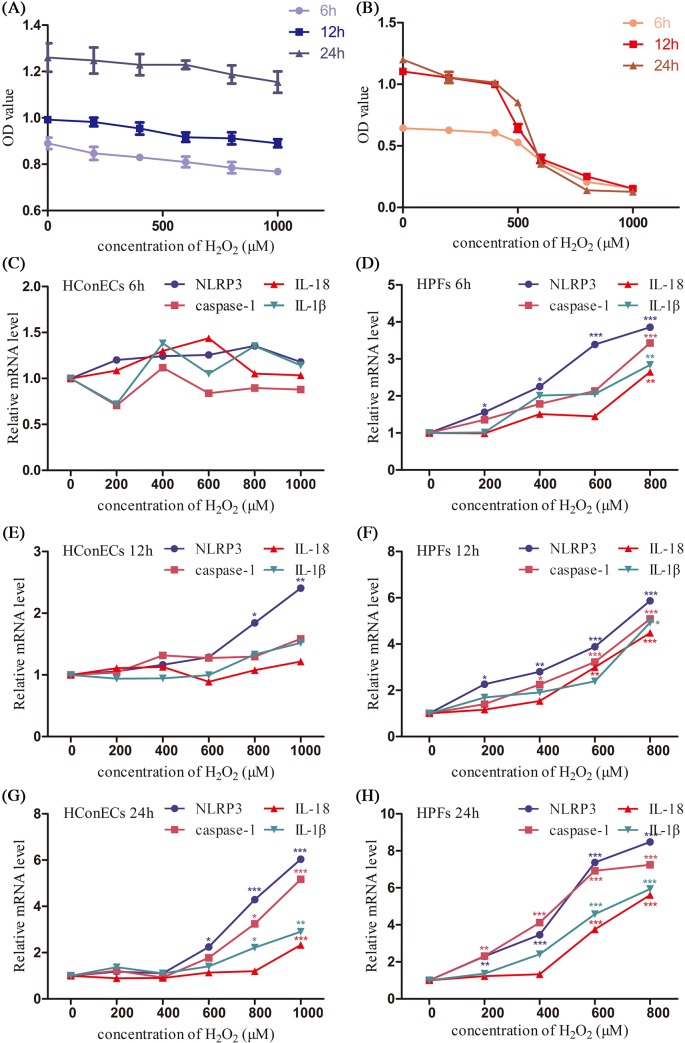
Comparison of sensitivity to H_2_O_2_ in HConECs and HPFs (1) (**A**) CCK-8 assay result of HConECs after co-culture with various concentrations of H_2_O_2_ for 6, 12, and 24 h; (**B**) CCK-8 assay result of HPFs after co-culture with various concentrations of H_2_O_2_ for 6, 12, and 24 h; (**C** and **D**) expression of NLRP3, caspase-1, IL-18, and IL-1β mRNA levels in HConECs and HPFs after co-culture with various concentrations of H_2_O_2_ for 6 h; (**E** and **F**) expression of NLRP3, caspase-1, IL-18, and IL-1β mRNA levels in HConECs and HPFs after being treated with different concentrations of H_2_O_2_ for 12 h; (**G** and **H**) expression of NLRP3, caspase-1, IL-18, and IL-1β mRNA levels in HConECs and HPFs after stimulation of H_2_O_2_ for 24 h; ^*^P<0.05, ^**^P<0.01, and ^***^P<0.001 compared with control.

**Figure 9:**
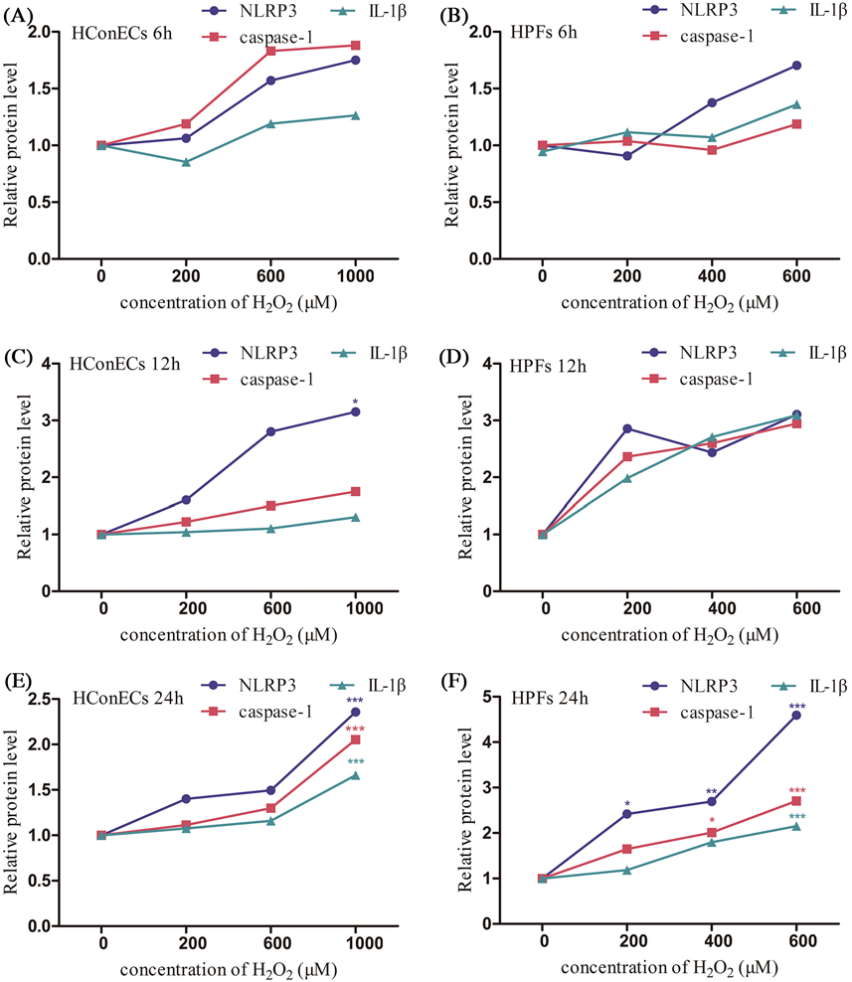
Comparison of sensitivity to H_2_O_2_ in HConECs and HPFs (2) (**A** and **B**) Expression of NLRP3, caspase-1, and IL-1β protein levels in HConECs and HPFs after co-culture with various concentrations of H_2_O_2_ for 6 h; (**C** and **D**) expression of NLRP3, caspase-1, and IL-1β protein levels in HConECs and HPFs after co-culture with different concentrations of H_2_O_2_ for 12 h; (**E** and **F**) expression of NLRP3, caspase-1, and IL-1β protein levels in HConECs and HPFs after co-culture with various concentrations of H_2_O_2_ for 24 h; blot images can be found in Supplementary Figure S1; ^*^P<0.05, ^**^P<0.01, and ^***^P<0.001 compared with control.

According to CCK-8 assay, the viability of HPFs was much more susceptible to H_2_O_2_ inhibition in comparison with HConECs under the same oxidative stress condition (Figure 8A,B). qRT-PCR, Western blot analysis, and immunostaining were performed to detect the changes of NLRP3, caspase-1, IL-1β, and IL-18 expression after being treated with various concentrations of H_2_O_2_ for 6, 12 and 24 h, respectively. (Figure 8C–H and Figure 9A–F, blot images are demonstrated in Supplementary Figure S1). NLRP3 elevation appeared in the lower concentration and shorter stimulation period in both types of cells, the phenomenon coordinated with the initiation function of NLRP3 in pyroptosis. The expression of NLRP3, caspase-1, IL-18, and IL-1β all demonstrated a dose-dependent elevation pattern related to the concentration of H_2_O_2_ as well as the stimulation period. The rise of pyroptosis factors occurred in HPFs at a relatively low H_2_O_2_ concentration and in a remarkably early stage during co-culture. Additionally, pyroptosis factors were notably increased in HPFs at certain concentrations and during periods of H_2_O_2_ stimulation, compared with HConECs exhibiting the same oxidative damage.

## Discussion

Pyroptosis was observed in 1992 as Caspase-1-programed cell death and was later termed in 2001 as to differentiate it from the morphologically distinct apoptosis. The pyroptosis effector gasdermin D was discovered in 2015 as a cleavage target for caspase-1 and caspase-11 [26]. Previous study showed that pyroptosis participates in many pathological processes. By validating the elevated pyroptosis-related factors in pterygium tissues and HPFs, we deducted the possible role of pyroptosis in pterygium pathogenesis.

To elucidate the mechanism of pyroptosis in pterygium genesis, we performed further investigations. Epidemiological evidence suggests that in pterygium genesis, UV irradiation plays the most important role. UV radiation to HConECs and HPFs results in the generation of ROS [27] and causes damage in DNA, proteins, and lipids [28], which may lead to the onset and progression of pterygia. The accumulation of ROS can also up-regulate the expression of some DAMP molecules that render inflammation [29]. For pyroptosis, there is evidence that activation of ROS generation can induce NLRP3 and caspase-1 activation to trigger IL-1β as well as IL-18 production during pyroptosis [30]. Our previous study showed that pyroptosis also participated in the oxidation of human lens epithelial cells. Previous studies hinted that the accumulation of ROS can lead to pyroptosis, which may participate in the pathogenesis of pterygia. H_2_O_2_, as a source of activated oxygen, is commonly used for *in vitro* cellular oxidative damage models because of its ability to permeate cellular membranes and enter the cells to cause injury [31]. We chose H_2_O_2_ to simulate the oxidative injury in pterygium genesis.

We proved that in HConECs, NLRP3, caspase-1, IL-1β, and IL-18 expression was increased after treatment with H_2_O_2_, and cell lysis, characterized by LDH release, which could be inhibited by cytoprotective agent glycine, was induced. Pyroptosis, with the characteristic of rapid plasma-membrane rupture and release of proinflammatory intracellular contents, was observed in H_2_O_2_-stimulated HConECs. This phenomenon indicates that H_2_O_2_ can cause increased cell permeability and fragility, and pyroptosis is induced afterward [32]. The caspase-1 inhibition experiment confirmed that caspase-1-dependent pyroptosis was induced in HConECs. These results indicate that oxidative stress participates in pterygium formation by initiating pyroptosis of HConECs.

To further explore the relationship between pyroptosis and pterygium progression, we analyzed the expression of pyroptosis-related factors in HPFs after treatment with H_2_O_2_ and a similar result is discovered in HPFs. Pyroptosis-related factors are significantly up-regulated in HPFs after H_2_O_2_ stimulation and downstream factors can be restrained after caspase-1 inhibition. Moreover, we discovered that pyroptosis can be induced in HPFs with a lower concentration of H_2_O_2_ in a shorter period of time, which indicates that patients with pterygia are more susceptible to oxidative stress, and pyroptosis is prone to be induced in HPFs. Pyroptosis is easily initiated once the pterygium has formed and large amount of proinflammatory cytokines are released.

NLRP3 is the initiating pyroptosis factor responding to certain cellular stress signals triggered by PAMPs and DAMPs and may activate caspase-1, thus initiating the process of pyroptosis [33]. The common causes of pterygia, such as UV radiation and other types of oxidative stress, can perform as DAMPs and trigger the process of pyroptosis. Caspase-1 plays an irreplaceable role in the process of pyroptosis, functioning as precursors of the inflammatory cytokines IL-18 and IL-1β [34]. IL-18 is a proinflammatory cytokine that participates in many biological processes, including cell fibrosis and epithelial-mesenchymal transition [35,36]. IL-1β is another pyroptosis-related cytokine that acts as an important mediator in inflammatory response and can be involved in all sorts of cellular activities, including cell proliferation, differentiation, apoptosis, EMT, and fibrosis [37]. After the occurrence of pyroptosis, IL-18 and IL-1β are activated and released in large numbers. Functional IL-18 and IL-1β cause all sorts of changes, such as fibrosis, EMT, apoptosis, and abnormal cell proliferation, all of which are included in mechanisms of pterygium genesis. Therefore, the elevation of these pyroptosis factors in HPFs, which is the main component of pterygia, may play a considerable part in the progression of pterygia.

The elevation of α-SMA and decrease of E-cadherin were observed in HPFs accompanied by H_2_O_2_-induced pyroptosis and both changes could be restrained by caspased-1 inhibition. The evidence suggests that pyroptosis in HPFs may lead to the transition of fibroblasts into activated myofibroblasts that accelerates fibroproliferation and extracellular matrix accumulation in pterygium progression. Apart from an identification of myofibroblast activation, α-SMA can produce fibrovascular tissue with higher density and solidity and lead to even severe pterygia [38]. The loss of E-cadherin, an intercellular adhesion molecule, in stimulated HPFs is another indicator of myofibrolasts activation and may also result in a more invasive growth pattern of HPFs [39]. Therefore, the pyroptosis in HPFs induced by H_2_O_2_ stimulation can give rise to the activation of myofibroblasts and the progression of pterygium.

Pyroptosis, accompanied by apoptosis and autophagy, has gained accumulating attention in recent years due to its programed cell death function in multiple biological and pathological processes. Evidence shows that multiple types of cell death are observed simultaneously in cells or tissues exposed to the same stimulus, suggesting that distinctive modes of cell death occur in an overlapping pattern after certain types of damage [40]. However, the precise mechanism of programed cell death interaction in response to a particular stimulus remains unclear. Apoptosis has been reported in pterygium pathogenesis associated with oxidative stress [41]. Thus, the functions and cross-linking reactions of apoptosis and pyroptosis in ROS-induced pterygium formation provide us an intriguing target in future study.

In summary, our results provided the first evidence that pyroptosis participates in the oxidation of HConECs and HPFs and may be involved in the pathogenesis of pterygia. The salient findings from the present study indicating the essential role of pyroptosis in pterygium formation and progression provide us a better understanding of pterygia and may unveil an innovative approach in pterygium treatment which would contribute to the reduction of medical burden worldwide.

## Supporting information

## Abbreviations

α-SMA: alpha-smooth muscle actin
CCK-8: cell counting kit-8
DAMP: damage-associated molecular pattern
DMEM/F12: Dulbecco’s modified Eagle’s medium/F12
DMEM/low glucose: Dulbecco’s modified Eagle’s medium/low glucose
EMT: epithelial-mesenchymal transition
FBS: fetal bovine serum
GAPDH: glyceraldehyde-3-phosphate dehydrogenase
HConEC: human conjunctival epithelial cell
HPF: human pterygium fibroblast
LDH: lactate dehydrogenase
NLRP3: nod-like receptor pyrins-3
PAMP: pathogen-associated molecular pattern
qRT-PCR: real-time quantitative polymerase chain reaction
ROS: reactive oxygen species
SEM: standard error of mean
