# Supplementary figures and images for: Pyroptosis in pterygium pathogenesis
Source: Biosci Rep. 2018 May 22;38(3):BSR20180282. doi: 10.1042/BSR20180282 (PMC6048216; doi:10.1042/BSR20180282)

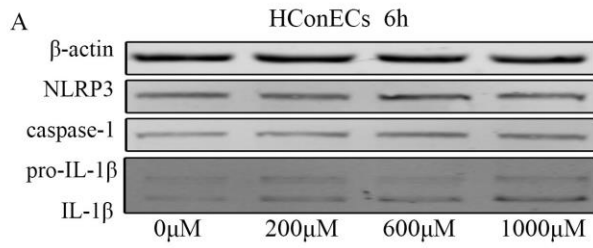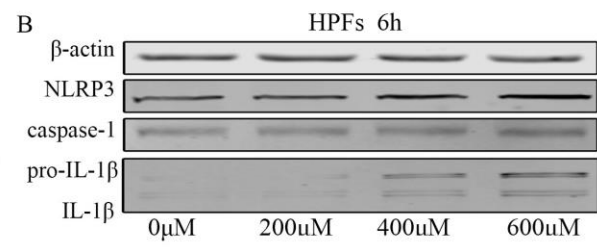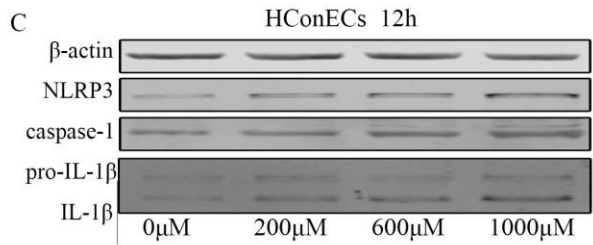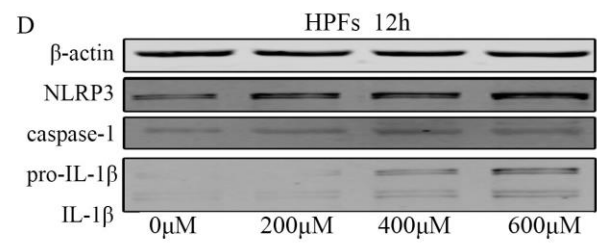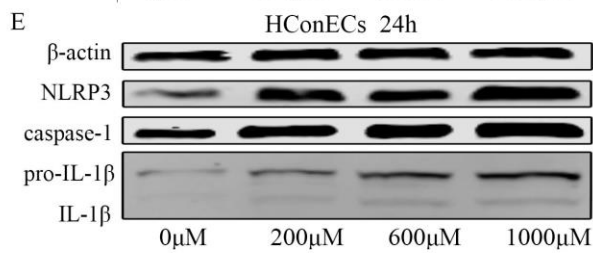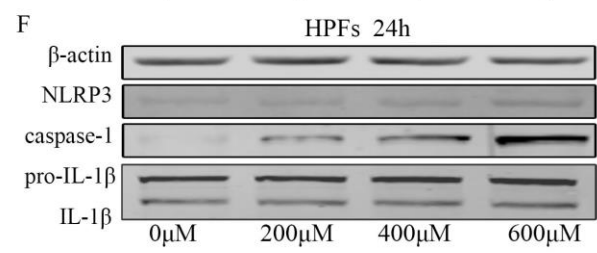

Supplement: Supplementary file 1 [file bsr20180282_Supp1.pdf]
